# Assessing volatile organic compounds exposure and prostate-specific antigen: National Health and Nutrition Examination Survey, 2001–2010

**DOI:** 10.3389/fpubh.2022.957069

**Published:** 2022-07-29

**Authors:** Chengcheng Wei, Yumao Chen, Yu Yang, Dong Ni, Yu Huang, Miao Wang, Xiong Yang, Zhaohui Chen

**Affiliations:** ^1^Department of Urology, Union Hospital, Tongji Medical College, Huazhong University of Science and Technology, Wuhan, China; ^2^Department of Urology, Ezhou Central Hospital, Ezhou, China; ^3^Department of Pathologist and Laboratory Medicine, Staff Pathologist, Deaconess Hospital, Evansville, IN, United States

**Keywords:** prostate-specific antigen (PSA), volatile organic compounds (VOCs), National Health and Nutrition Examination Survey (NHANES), public health, chloroform

## Abstract

**Background:**

Volatile organic compounds (VOCs) are a large group of chemicals widely used in people's daily routines. Increasing evidence revealed the VOCs' accumulating toxicity. However, the VOCs toxicity in male prostate has not been reported previously. Thus, we comprehensively evaluated the association between VOCs and prostate-specific antigen (PSA).

**Methods:**

A total of 2016 subjects were included in our study from the National Health and Nutrition Examination Survey with VOCs, PSA, and other variables among U.S. average population. We constructed XGBoost Algorithm Model, Regression Model, and Generalized linear Model (GAM) to analyze the potential association. Stratified analysis was used to identify high-risk populations.

**Results:**

XGBoost Algorithm model identified blood chloroform as the most critical variable in the PSA concentration. Regression analysis suggested that blood chloroform was a positive association with PSA, which showed that environmental chloroform exposure is an independent risk factor that may cause prostate gland changes [β, (95% CI), *P* = 0.007, (0.003, 0.011), 0.00019]. GAM observed the linear relationship between blood chloroform and PSA concentration. Meanwhile, blood chloroform linear correlated with water chloroform in the lower dose range, indicating that the absorption of water may be the primary origin of chloroform. Stratified associations analysis identified the high-risk group on the chloroform exposures.

**Conclusion:**

This study revealed that blood chloroform was positively and independently associated with total PSA level, suggesting that long-term environmental chloroform exposure may cause changes in the prostate gland.

## Introduction

Volatile organic compounds (VOCs) are a large group of chemicals used as solvents, degreasers, and cleaning agents through industry and consumer products in people's daily routines (1, 2), including vehicle emissions, cooking, wood burning, various industrial processes, smoking, cleaning supplies, building materials, and other household products (3–5). People monitored human exposure to VOCs through a variety of mechanisms, including traditional routes of assessment and the collection of an individual's blood, urine, breath, or sweat (6).

Previous studies have reported that acute exposures to selected VOCs [1,1-dichloroethane, 1,2-dichloroethane, benzene, bromodichloromethane, bromoform, chloroform, dibromochloromethane, dichloromethane, ethylbenzene, MTBE, tetrachloroethylene (PCE)] lead to kidney damage, cardiovascular disease, leukemia and chromosomal mismatch (7–9). Some studies showed that severe VOCs exposures [benzene, toluene, ethylbenzene, and xylenes (BTEX)] could cause sharp liver injury in humans and animal models and lower environment VOCs exposures may also lead to liver damage (10, 11). Some studies indicated that occupational VOCs (methane, benzene, toluene, ethylbenzene, o-xylene, m/p-xylene, and styrene) levels affect white blood level growth. Meanwhile, this phenomenon also occurs in the general population (4, 12, 13). Increasing evidence revealed the VOCs' accumulating toxicity (14). A study observed that cancer incidence, including stomach, bronchus, lung, and prostate, increased among persons living near a municipal solid waste landfill site that generated many VOCs (15). Although most research used empirical data in the environmental distribution and risk analysis, personal laboratory data was considered the best similar to actual exposure (16, 17). The limitations of the association between the blood VOCs concentration and parameters of liver, kidney, hematologic, endocrine and prostate functions still exist, especially in the average population (18). Studies evaluated occupational exposure to monocyclic aromatic hydrocarbons (MAHs) in prostate cancer (PCa) development indicating elevated risks of PCa with ever exposure to toluene and xylene (BTX) and duration, while marginal increases were found for exposure to styrene or MAHs (19–21). The limitations of the association between the blood VOCs concentration and parameters of liver, kidney, hematologic, endocrine, and prostate functions still exist, especially in the average population (18, 22).

The prostate is an essential reproductive gland in many physiological functions and fertility; it produces prostate fluid containing different enzymes, zinc, and many acids (23, 24). The prostate-specific antigen is one of the enzymes in a prostatic fluid whose functions include coagulation and liquefaction of semen which play a significant role in sperm fertility (25). Mounting PSA levels with an enlarged prostate size is related to benign prostatic hyperplasia (BPH), prostatitis, or prostate cancer (26). PSA concentrations monitoring is considered the most helpful serum biomarker to detect in the early prostate cancer process, clinical staging, and therapeutic outcome observation (27). Now, increasing evidence shows various environmental pollution like agent orange, pesticides, and cadmium are possible risk factors in prostate cancer (28), evidenced by PSA levels growth (29, 30). Air pollution and metal exposure are also known as risk factors for BPH like nitrogen oxide, cadmium, and nickel (31–33).

VOCs are common chemicals and suspected dangerous factors influencing public health. However, till now, there has been no research focus on the environmental VOCs affecting the prostate condition in the average population. However, some studies reveal that urinary volatiles and chemical characteristics for the non-invasive detection of prostate changes (34–37), the association between environmental VOCs exposure and PSA concentrations have not been reported previously. Studies revealed that occupational VOCs might promote PCa development, while environmental VOCs' effect on the prostate is still unclear. We hypothesized that VOCs might cause pathological prostate changes, which lead to PSA concentration changes. In order to verify our hypothesis, we explored the U.S. National Health and Nutrition Examination Survey (NHANES) for secondary analysis. According to previous articles (38–43), we controlled the potential confounders including, age, race, education level, marital status, poverty to income ratio, BMI, alcohol drinks, smoking, diabetes, physical activity, blood urea nitrogen, uric acid, creatinine which might be related to both the exposure as well as the outcome. Then we constructed models to clarify the comprehensive relationship between the volatile organic compounds (VOCs) and prostate-specific antigen (PSA). We aim to illustrate the VOCs influence prostate health among U.S. males.

## Methods

### Data availability

National Health and Nutrition Examination Survey (NHANES) as a nationwide study was supported by the National Centers for Disease Control (CDC) and Prevention National Health Statistics Center, which aimed to estimate the United States adults' and children's health and nutritional status from 1960 (44). All survey data and methodological details are available on the NHANES website (https://www.cdc.gov/nchs/nhanes/index.htm). NHANES protocols were approved by the NAHNES Institutional Review Board (IRB)/NCHS Research Ethics Review Board (ERB). In our study, no external IRB or ethical approval was needed beyond NHANES IRB/ERB approval.

### Study population

**Figure 1:**
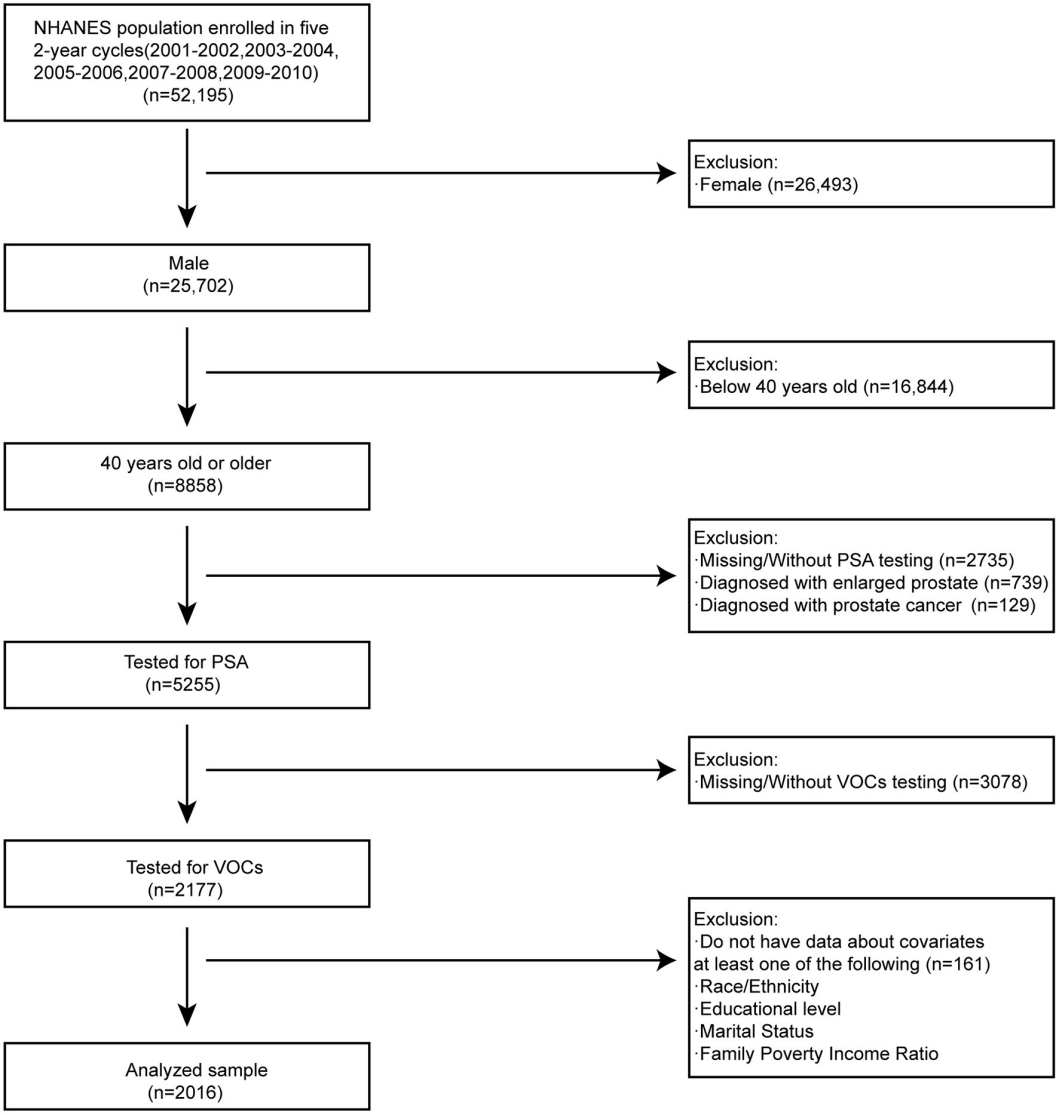
Flowchart for selecting analyzed participants.

NHANES is a continuous survey that has released published data files in 2-year cycles since 1999. Our study consisted of five periods of subjects who participated NHANES survey from 2001 to 2010. PSA concentration, water VOCs, blood VOCs, sociodemographic data, medical examination, and personal life history data, comorbidities data, and laboratory data have been included in our study for the secondary analysis. Participants were selected out of the total population and taken into our study according to the following exclusion criteria as: (1) female subjects (*n* = 26,493); (2) aged below 40 years old (*n* = 16,844); (3) missing/without PSA testing (*n* = 2735); (4) diagnosed with enlarged prostate (*n* = 739); (5) diagnosed with prostate cancer (*n* = 129); (Participants with enlarged prostate or diagnosed with PSA may cause outliers data which influence analysis results)(6) missing/without VOCs testing (*n* = 3078); (7) do not have data about covariates at least one of following (*n* = 161): race/ethnicity; educational level; marital level; family poverty income ratio (38, 43, 45). Inclusion criteria as: (1) male subjects (*n* = 25,702); (2) 40 years old or older (*n* = 8,858); (3) Tested for PSA (*n* = 5,255); (4) Tested for VOCs (*n* = 2,177); (5) have data about covariates at least one of following (*n* = 2,016): race/ethnicity; educational level; marital level; family poverty income ratio. There were 2016 analyzed samples out of 52,195 participants left in our study after screening (Figure 1). In the process of study design and conduction, the study complied with the Helsinki Declaration of the World Medical Association (46).

### VOCs measurement

Volatile organic compounds (VOCs) are a large group of chemicals that have been used as solvents, degreasers, and cleaning agents in the industry and consumer products. VOCs measurement consists of human blood VOCs and home tap water VOCs. Tap water VOCs including THMs (chloroform, bromodichloromethane, dibromochloromethane, and bromoform) and MTBE. These were analyzed by automatic method on headspace solid-phase microextraction (SPME) coupled with capillary gas chromatography and mass spectrometry. Blood disinfection by-products (DBP) (chloroform, bromodichloromethane, dibromochloromethane, and bromoform) and MTBE were quantified in human blood using capillary gas chromatography (GC) and high-resolution mass spectrometry (MS) with selected ion mass detection and isotope-dilution techniques which quantified trace levels of THMs and MTBE in human blood. Additional VOCs (tetrachloroethene, benzene, 1,4-dichlorobenzene, ethylbenzene, o-xylene, styrene, trichloroethene, toluene, m-/p-Xylene) were measured in human blood using SPME in conjunction with gas chromatography and benchtop quadrupole mass spectrometer.

### PSA measurement

Total PSA concentrations were detected using the Hybritech PSA method on the Beckman Access Immunoassay System, which automatically detected reacted samples' light production. Free PSA concentrations were detected by the Access Hybritech assay, which measures through a two-site immuno-enzymatic “sandwich” assay. Prostate-specific antigen ratio was calculated by dividing the free PSA by the total PSA and then multiplying by 100. Total PSA's cutoff value to dichotomize was 4.0 ng/ mL, and the PSA ratio was 15% (47, 48).

### Other variables

We have selected other variables affecting PSA concentration based on the previous surveys regarding the possible connection. Sociodemographic variables included age (year), poverty to income ratio, race/ethnicity (Mexican American, other Hispanic, non-Hispanic white, non-Hispanic black, others), an education level (less than high school, high school, more than high school), marital status (married, single, living with a partner). Variables of laboratory data included blood urea nitrogen (mmol/L), uric acid (umol/L), creatinine (umol/L) (41, 49, 50). The LX20 modular chemistry (BUNm) and timed endpoint method were used to quantitatively determine the concentration of blood urea nitrogen and uric acid in serum or plasma. Creatinine was detected by the LX20 modular chemistry side using the Jaffe rate method (kinetic alkaline picrate). Analyzed samples' comorbidities data included drinking (Had at least 12 alcohol drinks/1 year), smoking (Smoked at least 100 cigarettes in life), and diagnosis with diabetes (Yes, No, Borderline). Lastly, we also included medical examination and personal life history data involving body mass index (Kg/m2) and physical activity (MET-based rank). More details of variables can be found on the NHANES official website.

### Statistical analysis

We have conducted a statistical analysis of the VOCs (blood and water) and PSA levels based on the CDC guidelines' criteria (https://www.cdc.gov/nchs/nhanes/index.htm). PSA concentration, VOCs and other continuous variables were expressed as the mean ± standard deviation as the normal distribution. The categorical variables were presented as percentage or frequency. First, we divided age as a continuous variable into four quartile concentrations. The weighted chi-square was used to calculate the *p*-value of the characteristics of the analyzed population's Categorical variables. In the case of continuous variables, we used the Kruskal Wallis rank sum test to calculate the *p*-value. If the count variable has a theoretical number < 10, we used Fisher's exact probability test to calculate the *p*-value, Results were shown in Table 1. Second, we constructed the machine learning of the XGBoost algorithm model to predict the relative importance of blood VOCs on the effect of PSA concentration. XGBoost model was performed to analyze blood VOCs contribution (gain) to PSA concentration (51). Third, we constructed three kinds of weighted multiple linear regression models that adjusted various variables shown in Table 2 to classify the relationship between the blood VOCs and PSA concentrations (Model 1: non-adjusted model, Model 2: minimally adjusted model, Model 3: fully adjusted model). Multiple analysis results were based on Rubin's rules and calculated dataset. Then, we found the statistical difference between the blood chloroform and PSA level, so we further constructed the subgroup analysis to identify the stratified associations between blood chloroform and PSA through stratified multivariate logistic regression. Lastly, we based the penalty spline method to construct a smooth curve using a Generalized additive model (GAM) model with a fully adjusted model to explore the potential linear relationship between the blood chloroform and PSA concentration (52). In order to explore the origin of the blood chloroform, we further constructed a smooth curve between the blood chloroform and water chloroform. Using the MICE package accounting for missing data, we curated the data and improved the accuracy of analysis results (53). There is no significant difference between the complete data and origin data. Multiple analysis results were based on the calculated dataset and Rubin's rules. All kinds of statistical analyses were conducted by R software (Version 4.0.2) using the R package (http://www.R-project.org, The R Foundation) (54). The software of EmpowerStats provided significant help in the process of our research (http://www.empowerstats.com, X&Y Solutions, Inc., Boston, MA, USA). In our study, the level of statistical significance was set *p-*value below 0.05.

**Table 1:** Baseline characteristics of the study population.

**PSA, total**	**Q1**	**Q2**	**Q3**	**Q4**	***P*** **-value**
N	500	501	511	504	
PSA, total (ng/mL)	0.39 ± 0.12	0.73 ± 0.10	1.24 ± 0.22	4.01 ± 3.85	<0.001
PSA, free (ng/mL)	0.15 ± 0.06	0.24 ± 0.08	0.34 ± 0.13	0.84 ± 0.72	<0.001
Prostate specific antigen ratio (%)	38.37 ± 12.61	32.61 ± 10.88	28.11 ± 10.55	22.95 ± 9.82	<0.001
Sociodemographic variables					
Age, mean±SD (years)	54.38 ± 11.53	53.14 ± 10.92	56.07 ± 11.35	63.85 ± 11.76	<0.001
Poverty to income ratio, mean±SD	2.79 ± 1.64	2.80 ± 1.66	2.70 ± 1.64	2.79 ± 1.62	0.721
Race/Ethnicity (%)					0.128
Hispanic	111 (22.20%)	138 (27.54%)	128 (25.05%)	110 (21.83%)	
Non-Hispanic White	270 (54.00%)	253 (50.50%)	243 (47.55%)	259 (51.39%)	
Non-Hispanic Black	93 (18.60%)	96 (19.16%)	112 (21.92%)	111 (22.02%)	
Other race/ethnicity	26 (5.20%)	14 (2.79%)	28 (5.48%)	24 (4.76%)	
Education (%)					0.534
Less than high school	141 (28.20%)	151 (30.14%)	148 (28.96%)	168 (33.33%)	
High school	122 (24.40%)	132 (26.35%)	124 (24.27%)	120 (23.81%)	
More than high school	237 (47.40%)	218 (43.51%)	239 (46.77%)	216 (42.86%)	
Marital status (%)					0.028
Married	333 (66.60%)	341 (68.06%)	346 (67.71%)	317 (62.90%)	
Single	143 (28.60%)	125 (24.95%)	139 (27.20%)	169 (33.53%)	
Living with a partner	24 (4.80%)	35 (6.99%)	26 (5.09%)	18 (3.57%)	
Variables of laboratory data					
Blood urea nitrogen, mean±SD (mmol/L)	14.15 ± 5.52	13.77 ± 5.52	14.11 ± 5.06	15.69 ± 7.08	<0.001
Uric acid, mean±SD (umol/L)	6.18 ± 1.39	6.06 ± 1.26	6.11 ± 1.29	6.28 ± 1.37	0.043
Creatinine, mean±SD (umol/L)	1.03 ± 0.45	1.02 ± 0.46	1.06 ± 0.54	1.11 ± 0.47	<0.001
Comorbidities (%)					
Had at least 12 alcohol drinks/1 year?					0.357
Yes	379 (82.03%)	397 (84.83%)	402 (84.28%)	363 (80.67%)	
No	379 (82.03%)	397 (84.83%)	402 (84.28%)	363 (80.67%)	
Smoked at least 100 cigarettes in life					0.559
Yes	296 (59.20%)	295 (58.88%)	320 (62.62%)	300 (59.52%)	
No	204 (40.80%)	206 (41.12%)	191 (37.38%)	203 (40.28%)	
Diabetes					0.416
Yes	85 (17.00%)	65 (12.97%)	67 (13.11%)	82 (16.27%)	
No	404 (80.80%)	426 (85.03%)	436 (85.32%)	411 (81.55%)	
Borderline	11 (2.20%)	9 (1.80%)	8 (1.57%)	11 (2.18%)	
Medical examination and personal life history					
Body mass index, mean ± SD (Kg/m2)	29.77 ± 6.06	29.22 ± 5.82	28.32 ± 4.84	28.48 ± 5.32	<0.001
Physical activity (MET-based rank) (%)					0.514
Sits	276 (70.41%)	271 (68.61%)	286 (73.33%)	263 (76.01%)	
Walks	68 (17.35%)	75 (18.99%)	68 (17.44%)	46 (13.29%)	
Light loads	29 (7.40%)	31 (7.85%)	21 (5.38%)	21 (6.07%)	
Heavy work	19 (4.85%)	18 (4.56%)	15 (3.85%)	16 (4.62%)	
Water VOCs					
Water Bromoform, mean ± SD (ng/mL)	1.50 ± 3.43	1.54 ± 3.13	1.55 ± 3.74	1.85 ± 5.14	0.476
Water Chloroform, mean ± SD (ng/mL)	13.07 ± 18.24	14.31 ± 18.82	14.67 ± 19.78	12.63 ± 17.37	0.245
Water Bromodichloromethane, mean ± SD (ng/mL)	8.98 ± 13.26	8.19 ± 11.03	8.05 ± 11.13	8.07 ± 11.31	0.557
Water Dibromochloromethane, mean ± SD (ng/mL)	3.35 ± 5.34	4.05 ± 6.79	3.69 ± 5.42	3.90 ± 6.35	0.295
Water MTBE, mean ± SD (ng/mL)	0.11 ± 0.49	0.12 ± 0.80	0.09 ± 0.11	0.08 ± 0.08	0.563
Blood VOCS					
Blood Tetrachloroethene, mean ± SD (ng/mL)	0.09 ± 0.49	0.07 ± 0.18	0.07 ± 0.23	0.06 ± 0.15	0.37
Blood Bromoform, mean ± SD (pg/mL)	2.21 ± 8.82	2.29 ± 12.40	2.64 ± 20.73	1.67 ± 3.34	0.018
Blood Bromodichloromethane, mean ± SD (pg/mL)	2.95 ± 4.08	3.30 ± 4.90	3.16 ± 5.06	3.01 ± 4.32	0.654
Blood Benzene, mean ± SD (ng/mL)	0.08 ± 0.12	0.07 ± 0.12	0.09 ± 0.15	0.06 ± 0.10	<0.001
Blood Chloroform, mean ± SD (pg/mL)	14.95 ± 35.36	14.32 ± 20.31	15.98 ± 25.91	14.38 ± 21.36	0.002
Blood Dibromochloromethane, mean ± SD (pg/mL)	2.13 ± 4.33	2.20 ± 3.91	2.22 ± 4.34	2.18 ± 3.60	0.284
Blood 1,4-Dichlorobenzene, mean ± SD (ng/mL)	1.08 ± 4.26	1.51 ± 9.79	1.60 ± 7.94	1.51 ± 7.16	0.692
Blood Ethylbenzene, mean ± SD (ng/mL)	0.05 ± 0.06	0.07 ± 0.31	0.10 ± 0.42	0.08 ± 0.48	0.01
Blood MTBE, mean ± SD (pg/mL)	8.87 ± 44.02	9.51 ± 34.54	7.70 ± 34.10	6.06 ± 33.86	0.197
Blood o-Xylene, mean ± SD (ng/mL)	8.04 ± 57.09	8.59 ± 36.36	12.17 ± 85.32	14.50 ± 181.15	0.015
Blood Styrene, mean ± SD (ng/mL)	0.05 ± 0.05	0.05 ± 0.05	0.07 ± 0.23	0.05 ± 0.04	0.033
Blood Trichloroethene, mean ± SD (ng/mL)	0.02 ± 0.05	0.02 ± 0.03	0.02 ± 0.04	0.02 ± 0.03	0.281
Blood Toluene, mean ± SD (ng/mL)	0.24 ± 0.37	0.23 ± 0.46	0.27 ± 0.50	0.22 ± 0.57	0.009
Blood m-/p-Xylene, mean ± SD (ng/mL)	0.17 ± 0.25	0.21 ± 0.90	0.31 ± 1.23	0.21 ± 0.90	<0.001

*Q1–Q4: Grouped by quartile according to the total PSA. Our data included PSA concentrations, Sociodemographic variables, Variables of laboratory data, Comorbidities data, Medical examination and personal life history, comorbidities data Water VOCs and Blood VOCs for the second analysis*.

**Table 2:** Multivariate weighted linear model analysis reveals the association between the blood VOCs and PSA concentration.

**Exposure**	**Model 1** β **(95% CI) P**	**Model 2** β **(95% CI) P**	**Model 3** β **(95% CI) P**
Blood VOCs			
Blood Tetrachloroethene	−0.139 (−0.522, 0.245) 0.47927	−0.074 (−0.438, 0.290) 0.68923	−0.079 (−0.442, 0.283) 0.66777
Blood Bromoform	−0.002 (−0.010, 0.007) 0.71939	0.001 (−0.007, 0.009) 0.83055	0.001 (−0.007, 0.008) 0.88882
Blood Bromodichloromethane	−0.001 (−0.025, 0.022) 0.90277	0.007 (−0.016, 0.029) 0.54772	0.010 (−0.013, 0.033) 0.40911
Blood Benzene	−0.623 (−1.450, 0.203) 0.13952	−0.058 (−0.883, 0.768) 0.89119	0.033 (−0.855, 0.921) 0.94213
**Blood Chloroform**	**0.005 (0.001, 0.009) 0.00782**	**0.007 (0.003, 0.011) 0.00019**	**0.007 (0.003, 0.011) 0.00019**
Blood Dibromochloromethane	0.006 (−0.021, 0.032) 0.68547	0.013 (−0.013, 0.038) 0.33540	0.015 (−0.010, 0.041) 0.24261
Blood 1,4–Dichlorobenzene	0.006 (−0.009, 0.021) 0.43682	0.000 (−0.014, 0.015) 0.95258	0.004 (−0.011, 0.020) 0.57235
Blood Ethylbenzene	−0.090 (−0.394, 0.214) 0.56140	0.018 (−0.273, 0.308) 0.90565	0.040 (−0.262, 0.342) 0.79429
Blood MTBE	−0.003 (−0.006, 0.000) 0.07231	−0.000 (−0.003, 0.003) 0.84867	−0.000 (−0.003, 0.002) 0.75679
Blood o-Xylene	0.000 (−0.001, 0.001) 0.94056	−0.000 (−0.001, 0.001) 0.94836	−0.000 (−0.001, 0.001) 0.88010
Blood Styrene	−0.164 (−1.209, 0.881) 0.75820	0.169 (−0.835, 1.172) 0.74176	0.073 (−0.937, 1.083) 0.88759
Blood Trichloroethene	−1.606 (−4.529, 1.317) 0.28174	−0.976 (−3.768, 1.816) 0.49327	−0.894 (−3.692, 1.905) 0.53134
Blood Toluene	−0.096 (−0.339, 0.147) 0.43788	0.017 (−0.219, 0.254) 0.88598	0.018 (−0.224, 0.260) 0.88570
Blood m-/p-Xylene	−0.052 (−0.177, 0.073) 0.41568	−0.008 (−0.128, 0.111) 0.88984	−0.001 (−0.124, 0.121) 0.98312
Water VOCs			
Water Bromoform	0.017 (−0.010, 0.044) 0.20826	0.008 (−0.018, 0.034) 0.52935	0.012 (−0.017, 0.041) 0.40627
Water Chloroform	0.001 (−0.004, 0.007) 0.66229	0.003 (−0.003, 0.008) 0.32078	0.001 (−0.006, 0.009) 0.72821
Water Bromodichloromethane	0.004 (−0.005, 0.013) 0.40601	0.003 (−0.006, 0.011) 0.53086	0.004 (−0.006, 0.014) 0.46444
Water Dibromochloromethane	0.016 (−0.002, 0.033) 0.08270	0.011 (−0.006, 0.028) 0.19207	0.016 (−0.004, 0.035) 0.11278
Water MTBE	−0.088 (−0.318, 0.141) 0.45088	−0.076 (−0.295, 0.143) 0.49621	−0.221 (−0.635, 0.194) 0.29709

*Model 1: Non-adjusted model adjusts for none. Model 2: Minimally adjusted model adjusts for age, race/ethnicity, education level, marital status and poverty to income ratio. Model 3: Fully adjusted model adjusted age, race/ethnicity, education level, marital status, poverty to income ratio, BMI (kg/m2), Had at least 12 alcohol drinks/1 year, smoked at least 100 cigarettes in life, diabetes, physical activity (MET-based rank) (%), blood urea nitrogen, uric acid, creatinine. Bold values means statistically significant*.

## Results

### Characteristics of selected population

Table 1 shows the baseline characteristics of the chosen population from the NHANES dataset (2001-2010), which is weighted distribution. Variables included sociodemographic variables, laboratory data, comorbidities, medical examination and personal life history, water VOCs, blood VOCs, and PSA data. We grouped the population by quartile according to the total PSA (Q1-Q4). We found the distribution of poverty to income ratio, race/ethnicity, education level, drinking situation, smoking situation, diabetes, and physical activity in four quartiles indicated no statistical difference with *p* values > 0.05, and prostate-specific antigen ratio, age, marital status showed statistical difference with *p* values < 0.05. The older population showed a higher level of total PSA concentrations, which accorded to previous papers. Compared with the various groups, VOCs including blood bromoform, blood benzene, blood chloroform, blood ethylbenzene, blood o-Xylene, blood styrene, blood toluene, blood m-/p-Xylene showed distribution difference with a statistical significance which may indicate exposure difference between four groups while blood tetrachloroethene, blood bromodichloromethane, blood dibromochloromethane, blood 1,4-dichlorobenzene, blood MTBE and blood trichloroethene showed no exposure difference. Water VOCs including water bromoform, water chloroform, water bromodichloromethane, water dibromochloromethane, and water MTBE showed no statistical difference in the four groups. In our research, non-Hispanic Whites were the main population, and the following were the non-Hispanic Black. (This race/ethnicity variable was derived from responses to the survey questions on race and Hispanic origin. Respondents who self-identified as “Mexican American” were coded as such regardless of their other race-ethnicity identities. Otherwise, self-identified “Hispanic” ethnicity would result in the code “Other Hispanic” variable. We used “Hispanic” category to replace “other Hispanic” and “Mexican American”. All other non-Hispanic participants would then be categorized based on their self-reported races: non-Hispanic white, non-Hispanic black, and other non-Hispanic race, including non-Hispanic multiracial).

### Using machine learning of XGBoost algorithm model to explore the VOCs' relative importance

**Figure 2:**
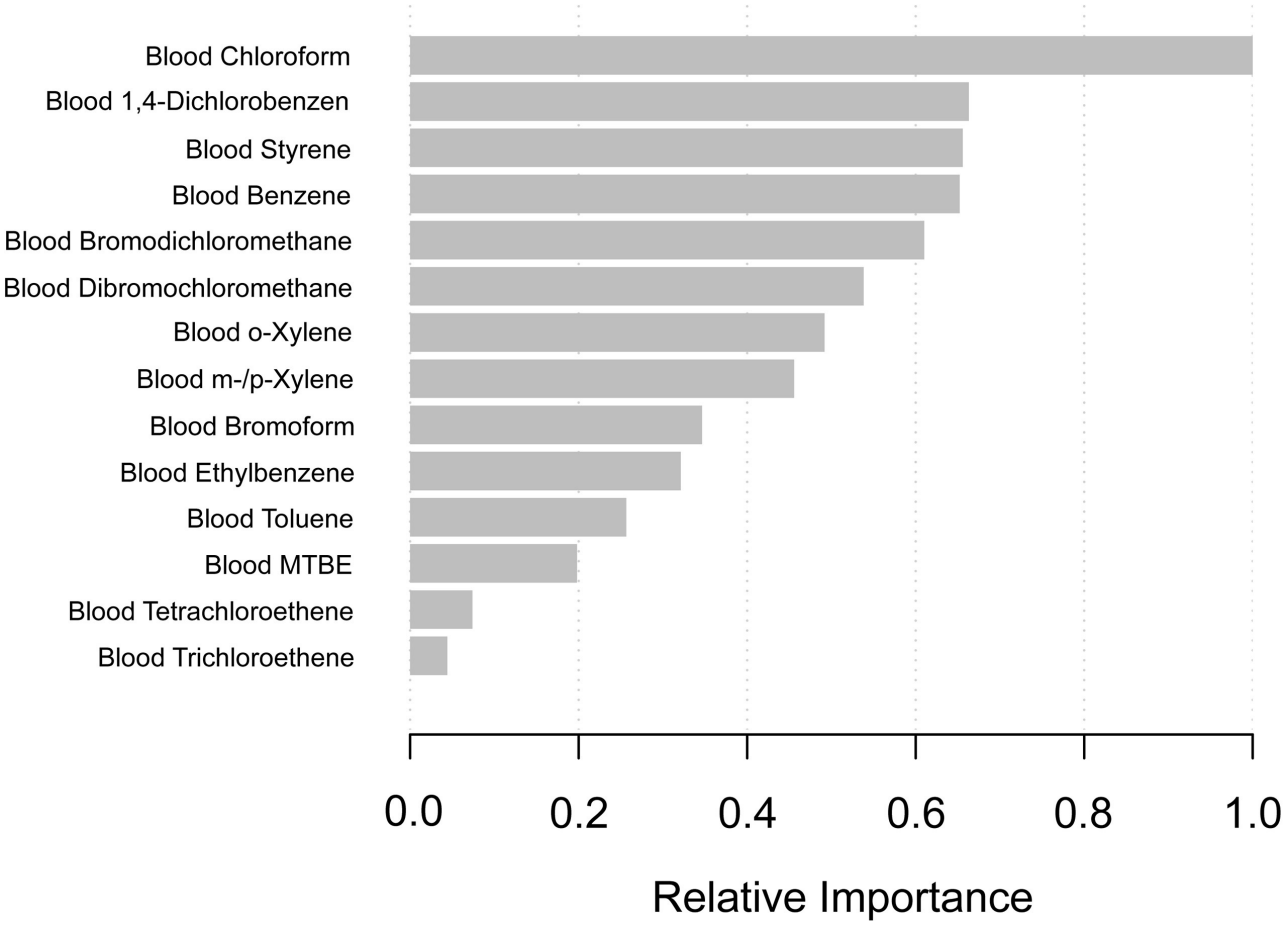
XGBoost model reveals the relative importance of blood VOCs on the PSA concentration and the corresponding variable importance score. The X-axis indicates the importance score, the relative number of a variable used to distribute the data; the Y-axis shows the blood VOCs.

In order to select which VOCs affected PSA most, we constructed the machine learning of the XGBoost Algorithm model to determine the relative importance among all blood VOCs. VOCs' variables included blood disinfection by-products (DBP) (chloroform, bromodichloromethane, dibromochloromethane, and bromoform) and additional blood VOCs (tetrachloroethene, benzene, 1,4-dichlorobenzene, ethylbenzene, o-xylene, styrene, trichloroethene, toluene, m-/p-Xylene). We found that blood chloroform was the most critical variable in the PSA concentration, followed by blood1,4-dichlorobenzene, blood styrene, blood benzene, and blood bromodichloromethane (Figure 2).

### The regression analysis between VOCs and PSA concentrations

In order to figure out the association between the blood VOCs and PSA concentrations, we constructed the weighted linear model by multivariate regression analysis shown in Table 2. Among all of the results, we found that only blood chloroform shows a positive association with PSA concentrations with statistical significance. In model 1 which adjusts for none, the PSA increase by 0.005 (ng/mL) (0.001, 0.009) for each additional unit of blood chloroform (pg/mL) with p less than 0.05. In model 2 (minimally adjusted model) and model 3 (fully adjusted model), results from both indicated that PSA increased by 0.007 (ng/mL) (0.003, 0.011) for each additional unit of blood chloroform (pg/mL) with p less than 0.05. This result suggested that long-time environmental chloroform exposure as an independent risk factor may cause male reproductive damage, especially in the prostate gland.

### Stratified associations between PSA concentrations and blood chloroform

We further analyzed stratified associations between PSA concentrations and blood chloroform in a specific subgroup by age, race, education level, and the ratio of family income and BMI shown in Table 3. Surprisingly, we found an association between the blood chloroform and the PSA concentration concentrated in the specific subgroup. PSA concentrations of the population whose ages from 60 to 70 increase by 0.050 (ng/mL) for each unit of blood chloroform in model 1, by 0.049 in model 2, and 0.060 in model 3 with *p* < 0.05. This positive association also represents the non-Hispanic black, education level more than high school, BMI from 25 to 28. In the non-Hispanic black, PSA increase by 0.036 (ng/mL) (0.025, 0.047) for each additional unit of blood chloroform (pg/mL) in model 1, increase by 0.035 (ng/mL) (0.024, 0.045) in model 2 and increase by 0.055 (ng/mL) (0.041, 0.070) with statistical difference. Moreover, people with education level more than high school had same trend whose PSA increase by 0.021(ng/mL) in model 1, increase by 0.022(ng/mL) (0.017, 0.028) in model 2 and increase by 0.027(ng/mL) (0.021, 0.033) in model 3 with statistical difference. Moreover, Population with BMI between 25 to 28 indicated that PSA concentrations increase by 0.009 (ng/mL) (0.004, 0.015) in model 1, increase by 0.011(ng/mL) (0.006, 0.016) in model 2 and 0.011(ng/mL) (0.005, 0.016) in model 3.

**Table 3:** Stratified associations of blood chloroform on PSA in the prespecified and exploratory subgroup.

**Blood Chloroform**	**N**	**Model 1** β **(95% CI) P**	**Model 2** β **(95% CI) P**	**Model 3** β **(95% CI) P**
Stratified by age				
<60	1170	0.000 (−0.002, 0.002) 0.8393	0.000 (−0.002, 0.002) 0.8041	−0.000 (−0.002, 0.002) 0.8647
**60–69**	**363**	**0.050 (0.035, 0.065) <0.0001**	**0.049 (0.034, 0.065) <0.0001**	**0.060 (0.040, 0.080) <0.0001**
70–79	292	0.005 (−0.021, 0.032) 0.6855	0.007 (−0.020, 0.034) 0.6012	0.001 (−0.060, 0.062) 0.9720
≥80	23	−0.043 (−0.113, 0.027) 0.2378	−0.016 (−0.111, 0.078) 0.7419	NA
Stratified by race				
Hispanic	445	−0.002 (−0.008, 0.003) 0.3444	−0.001 (−0.006, 0.004) 0.6431	−0.001 (−0.006, 0.004) 0.7159
Non-Hispanic White	940	−0.001 (−0.007, 0.005) 0.6943	0.002 (−0.003, 0.008) 0.4284	0.002 (−0.005, 0.009) 0.5653
**Non-Hispanic Black**	**3801**	**0.036 (0.025, 0.047) <0.0001**	**0.035 (0.024, 0.045) <0.0001**	**0.055 (0.041, 0.070) <0.0001**
Other race/ethnicit	83	−0.022 (−0.053, 0.009) 0.1745	−0.010 (−0.036, 0.016) 0.4362	−0.021 (−0.059, 0.018) 0.3109
Stratified by education				
Less than high school	554	−0.002 (−0.009, 0.006) 0.6716	0.000 (−0.007, 0.007) 0.9282	0.000 (−0.008, 0.009) 0.9153
High school	457	−0.001 (−0.008, 0.005) 0.6406	0.002 (−0.004, 0.008) 0.4545	0.005 (−0.004, 0.014) 0.2980
**More than high school**	**837**	**0.021 (0.015, 0.027) <0.0001**	**0.022 (0.017, 0.028) <0.0001**	**0.027 (0.021, 0.033) <0.0001**
Stratified by ratio of family income			
Low	608	−0.001 (−0.008, 0.005) 0.6947	0.000 (−0.006, 0.007) 0.9685	−0.000 (−0.008, 0.007) 0.9090
Middle	624	−0.004 (−0.013, 0.005) 0.3821	−0.001 (−0.009, 0.008) 0.8989	0.001 (−0.009, 0.010) 0.9047
**High**	**616**	**0.017 (0.011, 0.023) <0.0001**	**0.018 (0.013, 0.023) <0.0001**	**0.031 (0.024, 0.038) <0.0001**
Stratified by BM				
<25	409	−0.002 (−0.017, 0.012) 0.7515	0.001 (−0.013, 0.016) 0.8670	0.005 (−0.015, 0.026) 0.6111
25–28	467	0.009 (0.004, 0.015) 0.0006	0.011 (0.006, 0.016) <0.0001	0.011 (0.005, 0.016) 0.0001
>28	942	0.000 (−0.005, 0.005) 0.9477	0.002 (−0.003, 0.007) 0.3938	0.004 (−0.003, 0.011) 0.2411

*Note 1: Model 1: Non-adjusted model adjusts for none. Model 2: Minimally adjusted model adjusts for age, race/ethnicity, education level, marital status and poverty to income ratio. Model 3: Fully adjusted model adjusted age, race/ethnicity, education level, marital status, poverty to income ratio, BMI (kg/m2), Had at least 12 alcohol drinks/1 year, smoked at least 100 cigarettes in life, diabetes, physical activity (MET-based rank) (%), blood urea nitrogen, uric acid, creatinine*.

*Note 2: In each case, the model was not adjusted for the stratification variable itself. Bold values means statistically significant*.

### Linear relationship between the blood chloroform and PSA concentrations/water chloroform using GAM

**Figure 3:**
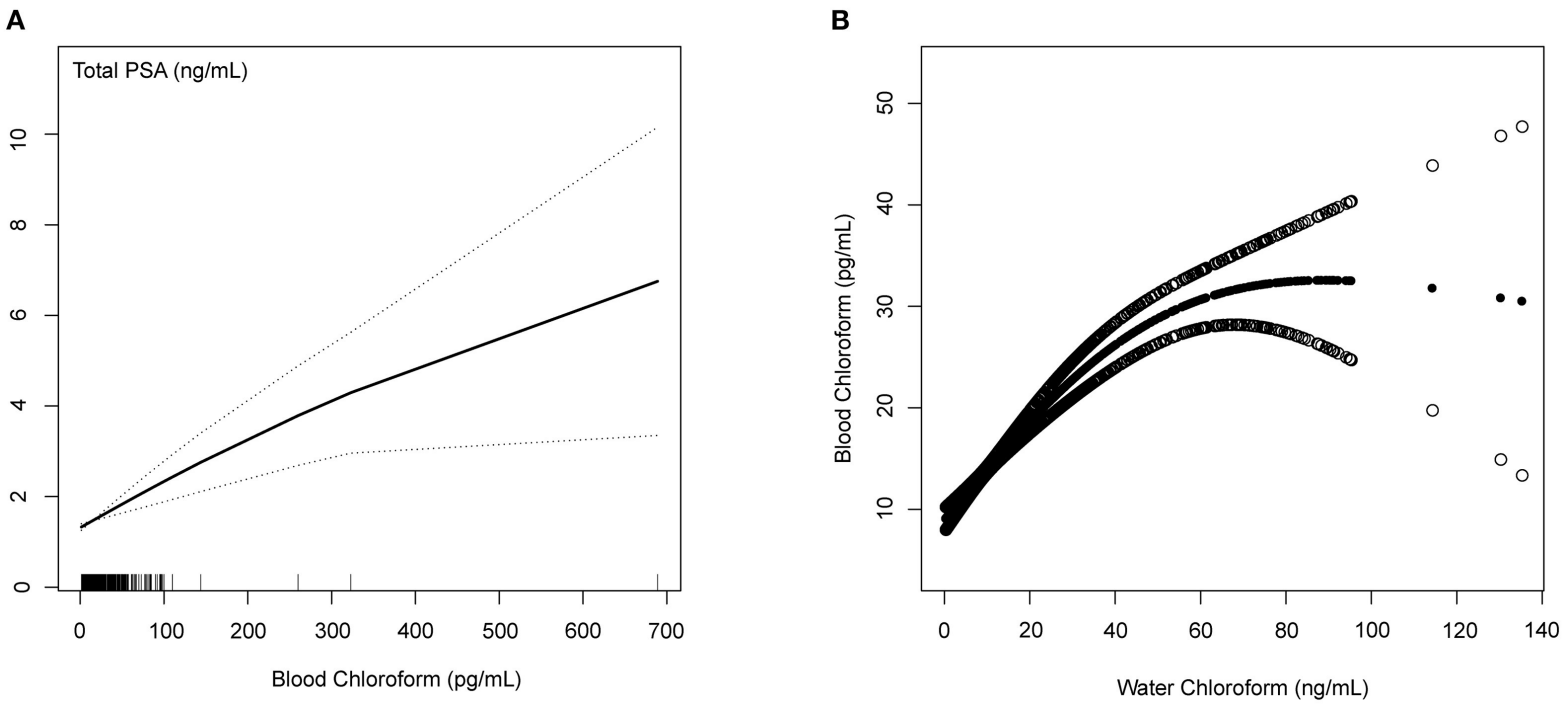
Blood Chloroform and PSA concentrations/Water Chloroform **(A)** The linear relationship between the blood chloroform and total PSA; **(B)** The nonlinear relationship between the blood chloroform and water chloroform.

The Generalized linear model (GAM) is sensitive to identifying the linear relationship or non-linearity. To confirm the stability of the analysis results, we constructed the linear relationship using the GAM model between the blood chloroform and PSA concentrations. Based on the fully adjusted model (Figure 3), we used a smooth fit curve to investigate the possible association. Adjusting for all variables, we observed the linear relationship between blood chloroform and PSA concentration, and most of the data were distributed in the blood chloroform between 0 to 100 (pg/mL). We also constructed the GAM model to explore the linear relationship between blood chloroform and water chloroform. We observed the linear relationship between the blood chloroform and water chloroform when the water chloroform concentration was below 40 (pg/mL). When water chloroform concentration was above 40 (pg/mL), we observed a non-linear relationship between the two variables, which indicated that the origin of blood chloroform might come from various sources when water chloroform is above 40 (pg/mL). Thus, this positive correlation suggested that absorption of water might be the primary origin of chloroform in the specific range.

## Discussion

VOCs serve as common chemicals in people's daily routines, including vehicle emissions, cooking, wood burning, various industrial processes, smoking, cleaning supplies, building materials, and other household products (55–59). More and more evidence revealed that VOCs might be the dangerous factors influencing public health. Although some studies reveal that urinary volatiles and chemical characteristics may help detect prostate changes in a non-invasive way (60–62), the association between environmental VOCs exposure and PSA concentrations has not been reported previously. Our research was an extensive secondary analysis of national studies to explore the potential relationship between volatile organic compounds (VOCs) and prostate-specific antigen (PSA) based on the United Stated cross-sectional NHANES database. We proposed the hypothesis that the toxicity of environmental VOCs might cause influent the function of the male reproductive gland. Until now, there is no previous epidemiological research that reported this association. Blood VOCs analysis was considered the accurate indicator used in the environmental pollution exposures assessment through toxic or harmful VOCs checked in human blood (63, 64).

VOCs in our study contained disinfection by-products (DBPs) (chloroform, bromodichloromethane, dibromochloromethane, and bromoform), MTBE, and other VOCs (tetrachloroethene, benzene, 1,4-dichlorobenzene, ethylbenzene, o-xylene, styrene, trichloroethene, toluene, m-/p-Xylene). DBPs are formed when chlorine interacts with natural organic materials found in water. Primary sources of DBPs mainly come from chlorinated drinking water and recreational water bodies (65, 66). DBPs showed the possibility of cytotoxicity, mutagenicity, teratogenicity, and carcinogenicity (67). Methyl-tert-butyl ether (MTBE) was used as an additive in gasoline to replace lead, but it was banned after widespread groundwater contamination was discovered (68). Other VOCs (tetrachloroethene, benzene, 1,4-dichlorobenzene, ethylbenzene, o-xylene, styrene, trichloroethene, toluene, m-/p-Xylene) usually used in industrial and chemical synthetic processes such as benzene has been used to produce DDT, phenol, and nitrobenzene, 1,4-dichlorobenzene is also used as a moth repellent and as a deodorizer (69). Some studies reported that occupational exposure to VOCs may correlate with cancers. A study indicated that blood THM species, particularly brominated THMs, were significantly associated with total cancer mortality in adults (70). A case-control study on occupational exposure to chlorinated solvents revealed elevated odds ratios (ORs) between perchloroethylene and prostate cancer (71). A study reported that cancer incidence increased among Finnish workers exposed to halogenated hydrocarbons (72).

Our research aimed to explore the toxicity of environmental VOCs exposures on the PSA level. We consisted of five periods of subjects who participated NHANES survey from 2001 to 2010 (2001-2002, 2003-2004, 2005-2006, 2007-2008, 2009-2010). In order to classify the above VOCs' relative importance on the PSA level, we first constructed the machine learning of the XGBoost model to determine the order of selected variables. We identified blood chloroform as the most important VOCs on PSA concentrations, followed by blood 1,4-dichlorobenzene, styrene, benzene, and bromodichloromethane. Then, we constructed a weighted linear model by multivariate regression analysis to identify which VOCs is the independent risk factor. Among all of the results, we found that only blood chloroform shows a positive association with PSA concentrations with a statistical significance which indicated that PSA increased by 0.007 (ng/mL) (0.003, 0.011) for each additional unit of blood chloroform (pg/mL) in model 3 (fully adjusted model). This result suggested that long-time environmental chloroform exposure as an independent risk factor may cause male reproductive damage, especially in the prostate gland. Results mean that if 200 (pg/mL) of blood chloroform is added, the PSA concentration will increase by 1.4 (ng/mL).

Chloroform is the most prevalent biomarker of DBPs which can be absorbed through ingestion, inhalation, and dermal contact (73). A prospective cohort study revealed a positive relationship between total brominated THMs, including Br-THMs, the sum of (BDCM, DBCM, and TBM) and TTHM concentrations, and the risk of cancer death (74). Some studies showed that long-term chloroform exposures are linked to colorectal cancer and bladder cancer (75, 76). Animal models indicated that lower exposures of chloroform causing maternal toxicity could not lead to offspring developmental effects (77, 78). Some studies also accounted for the association of chloroform with reproductive development outcomes at the human group level (79, 80). Till now, the toxicity effect of chloroform on the male reproductive gland has not been explored. Through constructing the Generalized linear model (GAM) model, we observed the linear relationship between blood chloroform and PSA concentration which indicated that the damage to the prostate is associated with the accumulation of chloroform exposures. Moreover, we observed the linear relationship between the blood chloroform and water chloroform when water chloroform is below 40 (pg/mL), indicating the absorption of water may be the primary origin of chloroform in the low dose range. Furthermore, we found that the association between chloroform on PSA level has population differences. We identified the high-risk group on the chloroform exposures through stratified analysis, including age between 60 and 70, BMI between 25 and 28, non-Hispanic black, education level more than high school, and education level more than high school.

Our survey has some limitations which should be acknowledged. First, although our study is national broad, most of the data is based on Unite State population. Data on Asian or other populations is still lacking, and the results of the VOCs exposures may be different due to the country's development. Second, this research is a cross-sectional design, and more research is needed to guarantee associations based on causal relationships. Third, VOCs had a relatively short half-life. Time differences may occur between the PSA concentrations and blood VOCs exposures. Although we comprehensively evaluated the association between VOCs exposure and damage to the prostate, was selected chloroform as a significant risk factor. Nevertheless, the number of analyzed subjects was still too small. It may be a deviation from the results. Therefore, the reproductive toxicity of VOCs and chloroform should be conducted in another large-scale study and *in-vivo/ in-vitro* experiments. In our research, we have excluded patients who contain factors that could affect PSA concentrations including diagnosed with enlarged prostate or with prostate cancer. However, our include patients may contain other factors that could affect PSA concentrations including the presence of prostatitis, drug treatment or recent prostate biopsy and surgery. Meanwhile, our study is based on the secondary analysis of published data, so variables that are not included in the data set cannot be adjusted for. With the development of VOCs consumption, it is necessary to monitor the VOCs concentration in serum and urine. We propose to construct more predicted models between VOCs and body biomarkers, including PSA, in the future clinical setting to guide clinical prevention and treatment.

## Conclusion

Our study comprehensively evaluated the association between VOCs exposure and serum PSA level. We found blood chloroform positively and independently associated with total PSA level, which suggested long-time environmental chloroform exposure may cause male reproductive damage, especially in the prostate gland. Furthermore, we found that blood chloroform positively correlates with water chloroform in the lower dose range, which indicated that the absorption of water might be the primary origin of chloroform. We also identified the high-risk group on the chloroform exposures. Our research wished to attract more attention to the toxicity of VOCs and prostate health among the average population.

## Data availability statement

The original contributions presented in the study are included in the article/supplementary material, further inquiries can be directed to the corresponding author/s.

## Author contributions

CW: conceptualization, data curation, formal analysis, methodology, software, visualization, writing-original draft, and writing-review & editing. YC: conceptualization, methodology, and writing-review & editing. YY: validation and writing-review & editing. DN, YH, and MW: writing-review & editing. XY and ZC: conceptualization, funding acquisition, methodology, supervision, and writing-review & editing. All authors contributed to the article and approved the submitted version.

## Conflict of interest

The authors declare that the research was conducted in the absence of any commercial or financial relationships that could be construed as a potential conflict of interest.

## Publisher's note

All claims expressed in this article are solely those of the authors and do not necessarily represent those of their affiliated organizations, or those of the publisher, the editors and the reviewers. Any product that may be evaluated in this article, or claim that may be made by its manufacturer, is not guaranteed or endorsed by the publisher.
